# Study on Optimization of High-Pressure Casting Process and Improvement of Mechanical Properties for Damping Spacer Based on ABAQUS

**DOI:** 10.3390/ma18184378

**Published:** 2025-09-19

**Authors:** Sen Jia, Anqin Liu, Kai Kang, Wenguang Yang

**Affiliations:** 1 School of Mechanical and Electrical Engineering, Yantai Institute of Technology, Yantai 264005, China; liuan_821@163.com (A.L.); kangkai@yitsd.edu.cn (K.K.); 2 School of Electromechanical and Automotive Engineering, Yantai University, Yantai 264005, China; yangwenguang@ytu.edu.cn

**Keywords:** spacer rod, mechanical properties, high-pressure casting, finite element simulation

## Abstract

A damping spacer rod is a key protective device in ultrahigh voltage transmission lines, which not only keeps the distance of split wires and limits the whipping and collision caused by the relative motion between sub-wires, but also inhibits the vibration of wires. This study aims to solve the problem of typical faults, such as loose wire clamps, that are prone to occur in damping isolation rods during long-term operation in ultra-high voltage transmission lines. Taking the spacer rod FGZ-450/34B as the object, a new high-pressure casting process for spacer rod frames is explored. The spacer rods were simulated by using the ABAQUS finite element software to predict the stress distribution and identify the dangerous sections. Based on this, the mold process was optimized to avoid die-casting defects. Meanwhile, mechanical property tests were carried out on the products produced by the two types of molds. The research finds that by optimizing the mold process, the die-casting quality of the dangerous section of the spacer rod can be effectively improved, and the best high-pressure die-casting scheme has been obtained through comparison. This research achievement provides technical support for enhancing the anti-vibration performance, anti-loosening reliability, short-circuit current thermal shock resistance, and anti-ultraviolet aging performance of damping isolation rods. It is of great significance for ensuring the stable operation of ultra-high voltage transmission lines and improving the production process level of damping isolation rods.

## 1. Introduction

With the promotion of the global energy interconnection strategy, UHV transmission lines have become the core carrier of trans-regional power transmission. A split wire system (typical 4/6/8 split structure) is a key technology for increasing transmission capacity, and its safe operation depends on the high-performance support of damping spacers [1]. As the core device for controlling the mechanical behavior of wires, the damping spacer has three functions: maintaining the standard distance of the sub-conductor, suppressing the breeze vibration and sub-span oscillation, and preventing the whiplash collision of the sub-conductor [2,3]. However, the traditional spacer rod has three failure modes: loosening of the clamp bolt, aging of the damping rubber pad, and breaking of the aluminum alloy frame. According to the statistics of State Grid in 2024, structural parts fracture accidents accounted for 63% of operation and maintenance faults [4,5].

The existing manufacturing process faces multiple challenges, such as shrinkage holes and cold insulation defects caused by the traditional gravity casting process, resulting in a stress concentration coefficient of the frame’s dangerous section as high as 2.3, which becomes the origin zone of fatigue cracks. At the same time, under a static load of 10 kN, the plastic deformation of traditional-process products is greater than 0.5 mm, which cannot meet the strict requirements of “pre-twisted fittings for overhead lines” on the rigidity of structural parts. In addition, the peak short-circuit current of the ±1100 kV line reaches 63 kA, which puts forward higher standards for materials’ thermal stability and the coordination of structural thermal expansion. Although a lot of research has been conducted to obtain spacers with excellent performance at home and abroad, most of the studies have only carried out a single simulation analysis or experimental analysis.

High-pressure die casting of ultra-large, thin-walled castings can hardly meet the high ductility requirements for structural applications [6,7,8]. Niu et al. aimed at ultra-large, thin-walled aluminum alloy castings, combined numerical simulation with experiments to study the influence of high-pressure die-casting process parameters on casting properties, defects, and mechanical properties [9]. They found that the yield strength of the casting slightly increases, while the elongation rate significantly decreases in the area far from the sprue, and they proposed the concept of effective flow length (EFL) to evaluate the casting properties. However, the inhomogeneous mechanical properties of die castings severely limit their application in structural scenarios that require excellent as-cast ductility. Zhang et al. studied the relationships among the process, microstructure, and mechanical properties of an AE44 magnesium alloy fabricated using high-pressure die casting (HPDC) under different process parameters (low-speed slow injection velocity, high-speed slow injection velocity, and solidification pressure) through three-dimensional reconstruction and two-dimensional characterization [10]. They found that the solidification pressure can enhance the feeding effect and reduce the porosity rate. Ma et al. utilized three-dimensional reconstruction and two-dimensional observation methods to explore the influence of different high-pressure die-casting parameters on the microstructure of castings [11]. The study found that when the rapid and slow injection pressure was not applied, large reticular shrinkage cavities would appear in the center of the sample. After pressure was applied to the casting, the melt filling was optimized, and the shrinkage cavities aggregated towards the center with a sharp decrease in their number. Meanwhile, the porosity (especially the gas porosity) near the gate was higher than that in the middle part of the rod-shaped sample. Lv et al. studied the synergy of a CRS, surface-hardening layer, and defect reduction, doubling the fatigue life [12]. In addition, a “ladder”-like mutation in the failure process was also observed, attributed to the resistance between pores and CRS.

To enhance the performance of extra-high voltage power accessories under harsh weather conditions while maintaining their structural integrity, Zhao et al. opted for high-strength and high-toughness aluminum alloys [13]. Employing finite element simulations, they systematically optimized the locations of the mold gates and runners to mitigate potential casting defects. Subsequently, mold modifications were implemented to facilitate vacuum die-casting, a process that further refined the casting quality and mechanical properties of the components. This approach not only addresses the practical challenges of weather resistance but also exemplifies a data-driven methodology for material and process optimization in high-voltage power applications.

The current research exhibits two critical limitations. First, it omits the utilization of finite-element simulations to pinpoint stress-concentration zones and forecast latent defects. Second, in the precision-casting phase, practical production and mechanical property validation rely on advanced casting technologies and equipment without leveraging digital-simulation-derived optimization, thereby undermining the synergy between theoretical analysis and experimental implementation.

To solve the above problems, this paper takes the FGZ-450/34B spacer rod as the research object and builds a closed-loop system of “digital simulation-precision casting-performance verification”. The finite-element model of the spacer rod was established via the ABAQUS software. Through simulation, the force analysis of the spacer rod frame is carried out, the dangerous cross-section location is discovered, and then the mold is modified.

Through digital simulation, the casting-process parameters are optimized in advance, effectively reducing the cost of trial and error and increasing the casting success rate. By utilizing high-precision mold-manufacturing technology, high-quality casting materials, strict process control, and dimensional accuracy, the surface quality and internal structure of the castings are ensured to meet high standards. A var of professional testing methods and equipment are employed to conduct comprehensive tests and evaluations on the physical properties, mechanical properties, and other key indicators of the castings. The performance verification results are fed back to the digital simulation stage and compared with the simulation prediction data for analysis, enabling the identification of model and casting process deficiencies. Based on the feedback information, the digital simulation model is corrected, and the precise casting-process parameters are optimized to form a complete closed loop. This iterative process continues, continuously improving the quality and performance of the castings and achieving high-quality development in product manufacturing.

## 2. Analysis of the Operating Mechanism of Spacer Rod FGZ-450/34B

**Figure 1:**
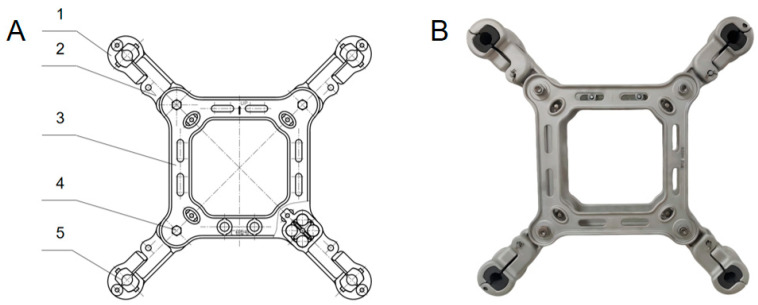
Structure diagram of spacer: (**A**) Assembly drawing. (**B**) Product structure diagram.

The spacer bar is mainly used to ensure that the spacing of split wire remains unchanged, thus ensuring the normal operation of the circuit, preventing the wire from short-circuiting, and suppressing wind vibration and shock. The FGZ-450/34B damping spacer introduced in this study adopts a four-grip elastic support structure. Schematic diagrams of the structural principle and the product structure are shown in Figure 1, where 1 denotes the clamp gland, 2 denotes the clamp body, 3 denotes the space frame, 4 denotes the cross shaft, and 5 denotes the rubber pad. A rubber pad is embedded in the grip, and through the compression of the rubber pad, a positive pressure is generated on the wire. The compression deformation of rubber compensates for wire creep, thereby eliminating wire vibration while gripping the wire. In addition, the support is connected with the body’s movable elastic joint to resist wire oscillation and torsion, so as to achieve damping and vibration elimination. When installing, use the tool to press the gland in place and insert the locking pin, eliminating the need for installing bolts and eliminating the risk of damage to the wire caused by the loosening of bolts by external forces or improper construction.

## 3. Finite-Element Simulation of Spacer Rod Force

In this research, the force analysis of the spacer frame was carried out using the ABAQUS 2024 simulation software, and the location of the dangerous cross-section was discovered, which provided a theoretical basis for subsequent research.

### 3.1. Spacer Rod Finite-Element Model Establishment

Set the splitting times of the damping spacer rod to be 4, the splitting distance to be 0.5 m, the self-weight to be 8 kg, and the splitting radius to be 0.319 m. ZL104 is selected for the frame, wire clamp theme, and cover plate, and its material properties are shown in Table 1.

The mesh type uses hexahedral elements. In the contact part, a modified second-order tetrahedral element C3D8 is used to accurately calculate the contact pressure.

**Figure 2:**
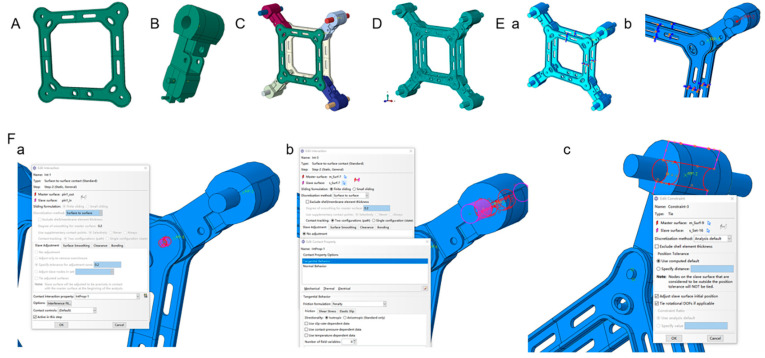
Finite-element model: (**A**) Spacer frame. (**B**) Pressing plates, damping pads, and coupling plates. (**C**) Assembly model. (**D**) Assembly model mesh. (**E**) Boundary condition–symmetry constraint. (a) Whole model. (b) Sub-model. (**F**) The connection between the components. (a) The connection between the frame and the main body of the cable clamp. (b) The connection between the pin shaft and the ball joint cover plate. (c) The main body of the cable clamp is bound to the ball joint cover plate.

The application of loads simulates the centripetal force on the spacer bar. The maximum centripetal force is applied as a concentrated load and coupled to the relevant contact surface. For the spacer bar model, a solid model is utilized. Specifically, the spacer bar frame, clamp body, clamp cover plate, and pin shaft are modeled separately and subsequently assembled, resulting in the overall geometric model of the four-division FGZ-450/34B spacer bar. Rigid connections are established among the frame, main clamp body, clamp cover plate, and pin shaft. The finite-element model of the FGZ-450/34B damping spacer is depicted in Figure 2.

### 3.2. Load Calculation

The centripetal force test of the spacer bar FGZ-450/34B frame was carried out to check whether the centripetal force of the spacer bar meets requirements. In accordance with the inspection requirements stipulated in DL/T1098-2016, this study conducted simulations of the spacer’s compression behavior under short-circuit conditions. Specifically, centripetal force was applied to the spacer, and by applying a concentrated load at the center of the conductor, a detailed analysis was performed on the mechanical properties of the spacer during short-circuit scenarios.

To apply the load, consider the safety margin of 1.2 times. In the case of *a* short-circuit, the spacer rod shall bear the centripetal force *P* of the wire on the structure:

(1) $$P=1.566\frac{2}{n}\sqrt{n-1}I_{cc}\sqrt{H\mathrm{lg}\frac{S}{D}}$$

where *n* is the number of sub-wires; *Icc* is the short-circuit current; *H* is the tension of the sub-conductor; *S* is the diameter of the sub-conductor splitting circle; *D* is the diameter of the sub-conductor. Equation (1) is derived from the requirements and tests for the overhead line spacer in the standard DL/T 1098-2016.

Through calculation, the centripetal force is 15.17 kN. According to the operation principle of the spacer rod, the load applied to the spacer rod is concentrated and coupled to the contact surface of the rubber tile, whose direction is along the spacer rod frame’s diagonal line. As shown in the calculation force in Figure 2, in order to facilitate analysis and save operation time, only a quarter of the frame structure is analyzed, and symmetric constraints are adopted in the middle section of the frame.

### 3.3. Analysis of Spacer Bar Simulation Results

The FGZ-450/34B damping spacer rod is modeled by using the C3D8R solid element. This element is an 8-node linear reduced integration three-dimensional solid element, which combines computational efficiency and solution accuracy [14]. It can effectively handle the mechanical behavior in scenarios with large deformations and large strains. It is widely applicable to contact problems and dynamic explicit analysis, which can accurately simulate the properties of composite materials and nonlinear materials, providing a reliable simulation solution for complex engineering problems.

**Figure 3:**
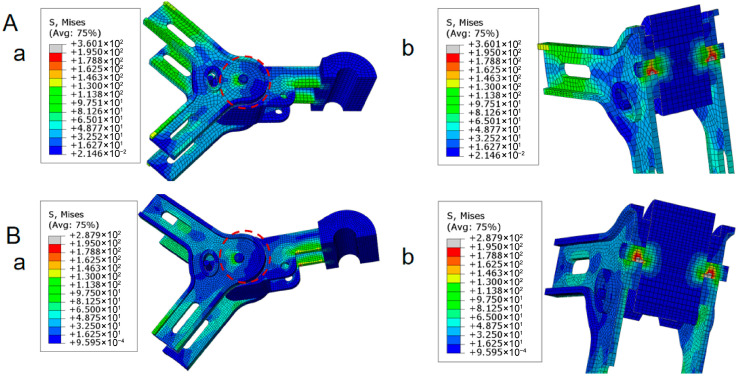
Finite-element analysis results of spacer bar under centripetal force condition: (**A**) Spacer rod stress cloud map with mesh size of 5 mm. (a) Whole model. (b) Section view. (**B**) Spacer rod stress cloud map with mesh size of 3 mm. (a) Whole model. (b) Section view.

The mesh size directly affects the calculation accuracy and efficiency. Reducing the mesh size can improve the simulation accuracy, but excessive refinement will significantly increase the computing time and even cause memory shortage problems. To explore the reasonable mesh size, the model was calculated using two different mesh sizes: 5 mm and 3 mm. The specific results are in Figure 3.

**Figure 4:**
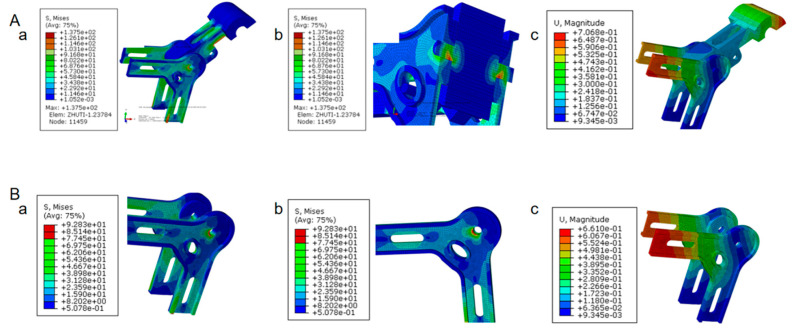
Finite-element analysis results of the spacer bar under the centripetal force condition: (**A**) Spacer rod stress and displacement cloud map with mesh size of 5 mm and 2 mm. (a) Whole model. (b) Section view. (c) Displacement. (**B**) Cloud map of spacer rod frame stress and frame displacement with mesh size of 5 mm and 2 mm. (a) Whole model. (b) Section view. (c) Displacement.

The simulation results indicate that, when a single mesh size is employed, local stress concentration emerges in the connection region, and the strength of certain meshes fails to satisfy the design specifications. The spacer bar structure has a minimum dimension of 5.1 mm. According to the meshing principles in numerical calculations, the region with this minimum dimension should theoretically be divided into at least two meshes, which corresponds to a mesh size of no more than 2 mm. During the attempt to perform calculations with a 2 mm mesh size, the combination of the structure’s complexity and the small mesh size resulted in an excessive number of generated meshes, and the computer’s memory capacity was exceeded due to excessive mesh refinement. In order to strike a balance between calculation accuracy and resource consumption, a non-uniform mesh-partitioning strategy was finally adopted. The overall model predominantly utilizes a 5 mm mesh size, while local mesh refinement is applied to critical connection areas. The mesh size in these refined regions is set at 2 mm. The optimized finite-element model of the JZF-45400 damping spacer is illustrated in Figure 4, which shows the finite-element model with a global mesh size of 5 mm for the main structure and a local refinement of 2 mm in the critical connection regions, along with the concurrently presented stress distribution contour plot and deformation results.

Under centripetal force experimental conditions, the root of the clamp’s main body is the dangerous area, whose maximum stress is 137.5 MPa, which is less than the allowable stress of the material, so as to meet the strength conditions (Figure 4A(a,b)). The maximum contact stress of the spacer bar frame reaches 92.83 MPa, occurring in the region where it makes local contact with the main body of the wire clamp. Subsequently, in the unoccupied area of the frame strip, the stress ranges from 41.95 to 76.41 MPa. The stresses in other areas are below 41.95 MPa, indicating relatively minor stress levels. The maximum overall displacement of the frame measures 0.699 mm, and this peak displacement is also observed in the unoccupied area of the frame strip, as clearly depicted in Figure 4B(a–c). From the above analysis, it can be seen that the long strip cavity area of the frame is a weak link, and it should be noted that there are no defects such as cold insulation, unsatisfactory pouring, or slag inclusion porosity in this area during the pouring process.

## 4. Material and Process of Spacer Rod FGZ-450/34B

### 4.1. Material

In order to reduce the force generated between the spacer rod and the wire, the lightweight of the device is particularly critical. Achieving lightweight levels requires a reduction in wall thickness while using lightweight materials. Aluminum alloy is the most widely used material for power fittings, whose specific strength (strength/density) is significantly higher than that of traditional steel, which can reduce weight by more than 60% under the same bearing capacity. On the other hand, the aluminum alloy surface oxide film (Al_2_O_3_) has the ability to resist atmospheric corrosion without additional galvanizing. Therefore, compared with steel fittings, the weight of aluminum alloy is reduced by 15–20%. In addition, the electrical conductivity of aluminum alloy is about 60% of that of copper, which meets the electromagnetic compatibility requirements of high-voltage transmission lines and avoids the increase in eddy current losses.

### 4.2. Ultra-High-Speed Die-Casting Process

The scientific basis for selecting LDPC, HDPC, and Squeeze Casting for comparative research lies in their typical representatives in terms of essential process characteristics, industrial applicability, and performance regulation dimensions, which can accurately align with the research objective of “optimization of the casting process and improvement of mechanical properties for damping spacers”. The inherent differences among the three processes in terms of molten metal filling mechanisms and pressure parameters (magnitude and timing) are directly associated with the evolution of internal defects (e.g., porosity, shrinkage porosity) and the morphology of microstructures in aluminum alloy damping spacers (featuring complex structures such as hollow cavities and reinforcing ribs). This further forms a critical action chain that influences their mechanical properties, which is precisely the core scientific issue to be analyzed in this study.

The machining technology of the spacer rod directly affects its mechanical properties. At present, the main production methods of the spacer frame include low-pressure die casting, high-pressure die casting, and extrusion casting. Low-pressure die casting (LPDC) refers to the slow filling of liquid metal in the mold under a low-pressure environment, which can effectively reduce turbulence and involved gas, resulting in fewer porosity defects and higher density of the castings. However, LPDC requires a control system and sealing molds with low pressure, and the initial investment is large. The filling and solidification time is longer, the single cycle is longer than that of high-pressure die casting, and the minimum wall thickness is still higher than that of high-pressure die casting.

Squeeze Casting (SC) is a method of filling a die-casting mold cavity with liquid or semi-liquid metal at a high speed under high pressure and forming and solidifying under pressure to obtain castings; SC has two major characteristics: high-pressure and high-speed filling of the die casting. This method has no runner system, low material loss, and a high material utilization rate. However, high-tonnage presses and dedicated molds are required, and the initial cost is significantly higher than that of die casting. The filling and holding time are long, and the single cycle is several times longer than that of die casting. In addition, the method is often used to manufacture simple or symmetrical structures, and it is difficult to form complex cavities, which cannot meet the design parting surface required for multi-branch spacers.

High-pressure die casting (HPDC) uses high pressure for high-speed filling. It is characterized by a short molding cycle, which is suitable for the large-scale production of spacer rods with a wall thickness of 0.5–1 mm. Moreover, the precision mold ensures that the surface of the casting is smooth, minimizing subsequent machining processes. It is capable of manufacturing intricate structures with a wall thickness ranging from 0.5 to 1 mm, thereby fulfilling the high-precision demands of lightweight design. However, high-speed filling is prone to involving gas, which forms subcutaneous pores and affecting the strength and corrosion resistance of the casting (requiring subsequent heat treatment or vacuum die casting improvement). The differences among LDPC, HDPC, and Squeeze Casting are illustrated in Table 2.

In conclusion, high-pressure die casting is particularly well suited for the mass production of lightweight spacer rods. In this study, a novel high-pressure die-casting process, the ultra-high-speed die-casting method, was employed. This innovative approach effectively mitigates the common defects associated with traditional high-pressure die casting.

**Figure 5:**
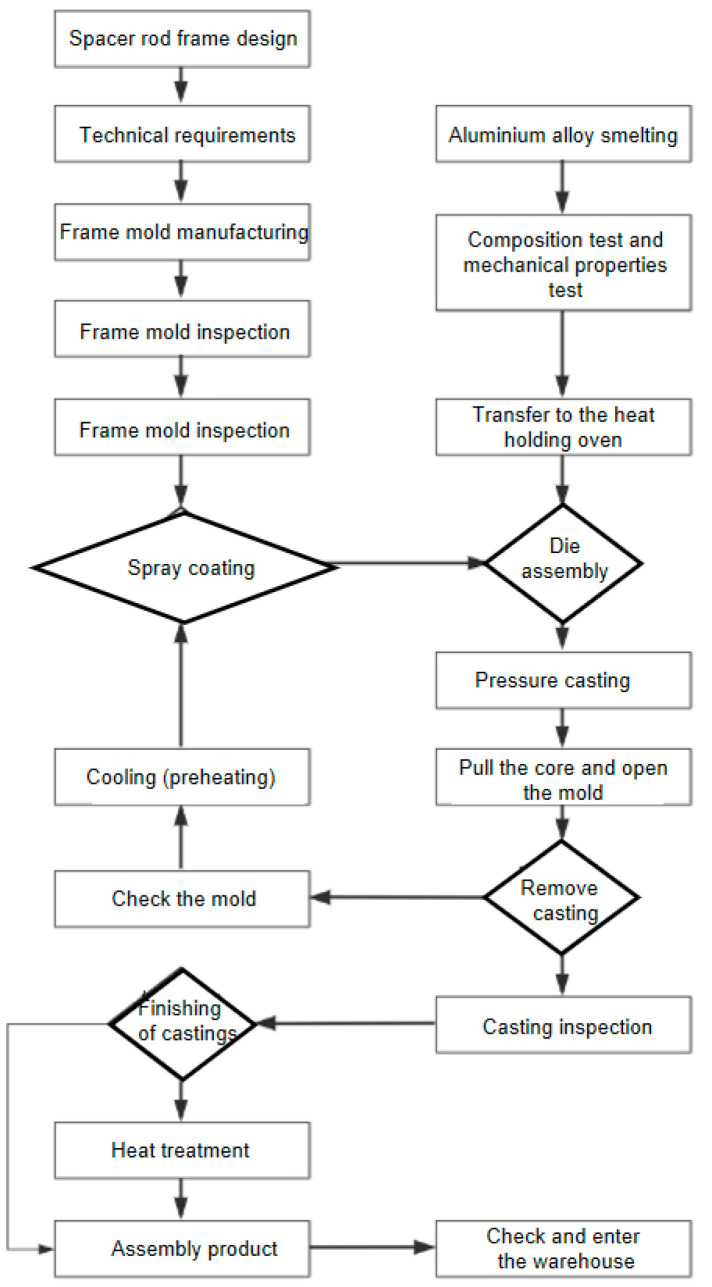
Spacer-die-casting process flow chart.

When liquid ZL104 is slowly filled into the mold, the casting temperature is controlled within the range of 650–680 °C, air in the mold cavity is discharged, and the die casting is carried out under rapid pressure, so that the liquid aluminum is cooled and solidified quickly to reduce the shrinkage phenomenon of the product. The specific process flow is shown in Figure 5.

During the die-casting process, Aluminum liquid in the solid–liquid two-phase region (aluminum water) is injected through the die-casting channel with a ladle. The punch is slowly fed to discharge the air in the mold cavity and then quickly pressurized for die casting, so that the liquid aluminum is cooled and solidified quickly to reduce the shrinkage phenomenon of the product. Finally, maintain the pressurization, ensure that the product is completely cooled, and release the pressure back to the punch. After the die casting is completed, open and close the die-casting mold and remove the die-casting product. It is worth noting that the die casting of the product should be properly maintained after the completion of the mold time (which, according to the product thickness, is generally 10 to 15 s).

During the die-casting process, the mold should be kept at a constant temperature to prevent the defects of flow marks, cold isolation, and insufficient pouring caused by the cooling and solidification of aluminum water in the flow process, resulting in the strength of the product at the cold isolation.

### 4.3. Problems and Solutions of Ultra-High Speed Die Casting

The instability of the flow state during high-pressure and high-speed filling can easily cause various defects, so the requirements for the pouring system are stricter. The improper pouring system will cause the gas to be discharged too late from the casting, which will affect the quality and application of the spacer thin-wall casting frame. The rational design of the die-casting gate system, including the rational design of the gate, runner, and overflow trough, will directly affect the quality of die casting.

**Figure 6:**
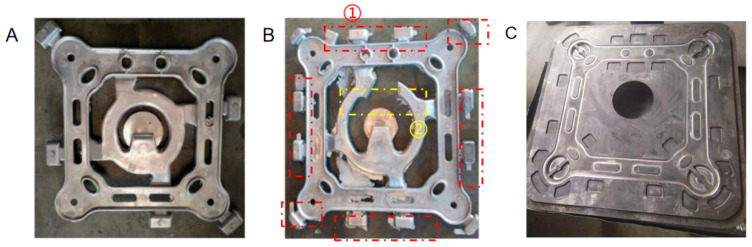
Spacer rod mold products. Red circles ① indicate that the structure of the overflow channel has been modified. The yellow circle ② indicates that the layout of the flow channel has been changed. (**A**) The spacer frame products produced by the initial die-casting mold. (**B**) The spacer frame products produced by the modified die-casting mold. (**C**) The modified casting mold.

**Figure 7:**
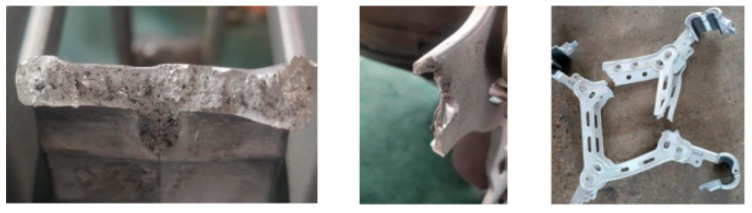
Result of the centripetal force experiment.

The spacer frame products produced by the initial die-casting mold based on this study are shown in Figure 6A. The tensile test found that the product does not meet the required product performance; the experimental section is shown in Figure 7. The primary reason lies in the structural characteristics of the FGZ-450/34B frame, which includes a large parting surface, thin arms, notably non-uniform wall thickness, and a high number of holes.

As shown in Figure 6A, the overflow channels are small in number, narrow in mouth, and long in flow path, which cannot discharge harmful inclusions, gases, and residues in the cavity in a timely and effective manner, resulting in defects such as cold isolation, insufficient pouring, and slag inclusion pores.

In response to the issues presented in Figure 6A, an optimized design for the mold was developed. The improvements, primarily consisting of two significant modifications, are clearly depicted in Figure 6B. The modified casting mold is shown in Figure 6C.

#### 4.3.1. Optimize the Structure Design of Overflow Channels

The structural design of the overflow channels was optimized based on the shape and dimensions of the mold cavity. The number of overflow channels was increased, with additional channels incorporated in regions prone to gas entrapment, inclusions, and residue accumulation—specifically, casting dead corners and transition zones between thick-walled and thin-walled sections—to enhance the efficiency of gas and residue evacuation. In detail, three overflow channels were added at the uppermost structural position farthest from the pouring gate, one overflow channel at the lowermost position closest to the pouring gate, and two overflow channels at the lateral structures between the middle positions of the framework. Additionally, one overflow channel was configured at each of the four intersection points of the framework.

Concurrently, the entrance width and effective volume of the overflow channels were increased, while the original rectangular cross-section was replaced with an appropriately sized semicircular configuration. This modification enhances the contact area between the overflow channels and the molten aluminum, facilitating smoother flow of the molten metal into the channels and enabling timely discharge and removal of harmful inclusions, gases, and residues retained within the cavity. The depth of the overflow channels was optimized to ensure sufficiency in preventing molten aluminum from spilling during the overflow process.

#### 4.3.2. Improve the Flow Channel Layout

The original structure featured an excessively long flow path, which failed to effectively and timely expel harmful inclusions, gases, and residues trapped within the cavity, thereby inducing defects such as cold shuts, incomplete filling, and slag pores.

Following optimization, the flow distance of molten aluminum within the channels was shortened, and the flow resistance was reduced. This modification enabled more direct entry of molten aluminum into the cavity while minimizing its residence time in the channels, consequently lowering the likelihood of gas entrainment and inclusion formation. Additionally, the channel geometry was optimized through the adoption of gradually varying cross-sections and smooth bends. These design improvements facilitated laminar flow of molten aluminum, avoiding turbulent conditions and vortex formation, which in turn reduced erosion of the cavity walls and mitigated the generation of oxide inclusions.

Based on the aforementioned analysis, and with comprehensive consideration of relevant technical factors and economic feasibility, this study modified the quantity, dimensions, and angles of the overflow grooves in the original die-casting mold. The resulting frame products are presented in Figure 6B. Centripetal force tests were conducted on the optimized products, with the corresponding results shown in Figure 7. These tests revealed that fracture initiation occurred at the root of the four gripping paws, which is consistent with the finite-element analysis results depicted in Figure 4, confirming that the performance requirements are satisfactorily met.

#### 4.3.3. Economic Analysis of Process Optimization Benefits

To evaluate the economic benefits of high-pressure die-casting process optimization, three production batches (10,000 pieces per batch, totaling 30,000 finished products) were selected as samples, and three key indicators were focused on, which are material utilization rate, scrap rate, and production cycle time. Then, the economic value of process optimization, through a comparative analysis between the optimized scheme and the original scheme, was quantified. The specific data comparison is shown in Table 3.

After process optimization, the production indicators of high-pressure die-casting achieved breakthrough improvements. The material utilization rate increased by 19.7%, significantly reducing raw material waste; the scrap rate decreased sharply by 78.2%, markedly enhancing quality stability. The production cycle per unit was shortened by 11 min, which means that, for each production batch of 10,000 products, the delivery can be advanced by 76 days. As a result, the production efficiency and delivery capacity have improved simultaneously, and the comprehensive economic benefits are remarkably significant.

### 4.4. Spacer Die Mechanical Properties Test

**Figure 8:**
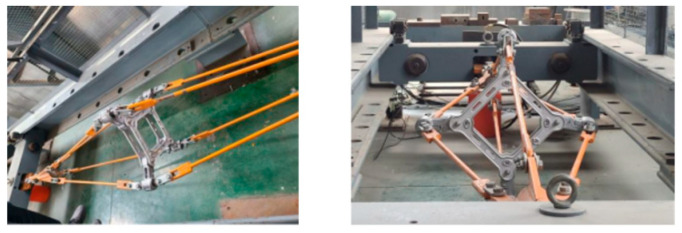
Centripetal force experiment.

According to the technical conditions and test methods of DL/T1098-2016, the sampling tension test of the spacer frame was carried out. An initial value of 15.17 kN was applied to the spacer rod to test its mechanical properties. The process is shown in Figure 8. The pulling force is applied in the horizontal direction, gradually increasing the pulling force, so that the centripetal force acting on the spacer rod reaches the specified value. Continue loading slowly until the spacer rod breaks and the experiment is over.

The test results of the mechanical product’s properties before and after mold modification are shown in Table 4.

It can be seen from Table 4 that the mechanical properties of products produced by the original mold cannot meet the requirements under the tensile test conditions. Due to the mold’s unreasonable design, serious defects, such as slag inclusion, porosity, and cold insulation, appear. The products produced after the modification of the mold meet the mechanical properties, and there are no obvious defects in the section when the main body of the clamp is broken and the frame is seriously deformed and broken. When the load reaches the limit value, the clamp root of the spacer rod breaks, and the clamp root is a weak link. When the wire clamp with high performance is selected, the long hole of the frame is broken, and the long hole is a weak link, which is consistent with the finite-element calculation results.

## 5. Conclusions

The spacer rod frame introduced in this study adopts ultra-high-speed die casting and slowly fills with rinsing aluminum water to discharge the air in the mold cavity. Through rapid pressure die-casting, the liquid aluminum is cooled and solidified quickly to reduce the shrinkage of the product. In addition, the original frame mold was modified. The quick-cut scheme is selected to increase the number of overflow channels, and its structure, shape, and size are modified to eliminate defects such as cold insulation, insufficient pouring, and slag inclusion pores from the source.

The finite-element simulation results of the spacer bar frame show that, under the tension test condition, the stress at the root of the main clip is the largest (the maximum value is 136.5 MPa), followed by the long hole area of the frame. Therefore, the bearing capacity near the root of the clamp and the long hole of the frame is relatively weak, and no defects can occur during the die-casting process.

Even if the same die-casting process is used, the products produced based on the original mold are limited by the number, size, and structural form of the overflow groove, and the mechanical properties cannot meet the requirements of the technical conditions, accompanied by serious defects, such as slag inclusion, porosity, and cold insulation. After the modification, the mechanical properties of the product easily meet the requirements of the technical conditions: the load exceeds the allowable load 1.2 times and continues to load, the main body of the clamp is broken, the frame is seriously deformed and broken, and the section has no obvious defects. The results show that a reasonable die design can solve the die-casting defects from the source and avoid the possibility of strength failure. The analysis provides a basis for the improvement of the spacer structure and production process, laying a solid foundation for the finite element simulation of spacers.

## Figures and Tables

**Table 1:** Mechanical properties of ZL 104 aluminum alloy.

Alloy Designation	Material Density (kg/m^3^)	Elasticity Modulus (GPa)	Poisson’s Ratio	Ultimate Strength (MPa)
ZL104	2650	69	0.34	195

**Table 2:** Principles, advantages, and disadvantages of process types.

Process Type	LPDC	HPDC	Squeeze Casting
Principle	Low-pressure filling, gravity solidification	High speed and high pressure filling, fast solidification	Liquid metal solidifies under pressure
Aluminum alloy grade	A356, A357 (Low silicon series)	ADC12, YL113 (High silicon series)	A2014, 6061 (high-strength system)
Typical application	Complex structures (e.g., articulated arms)	Thin-walled parts (wall thickness ≤ 3 mm)	High bearing parts (clamp body, joint shaft)
Advantage	Smooth filling and low porosity	High production efficiency (cycle < 30 s)	Grain refinement
Disadvantage	Cold insulation	Stomata	Crack

**Table 3:** Economic benefits.

Performance Indicator	Original Process	Optimized Process	Improvement	Remarks
Material Utilization (%)	66%	79%	+19.7%	Reduced weight
Defect Rate (%)	8.7%	1.9%	−78.2%	
Production Cycle Time (min)	49	38	−22.4%	Faster solidification

**Table 4:** Results of spacer bar experiment.

Experimental Project	Label	Standard Value of Centripetal Force (kN)	Centripetal Force Value (kN)	Test Tensile Force Standard Value/Measured Value (kN)	Damage and Condition
Original mold product (Old spacer frame/old clamp)	1	15.17	12.38	297.9 ± 9.6/243.2	Severe slag inclusion and porosity
	2	17.17	10.62	297.9 ± 9.6/208.6	Severe slag inclusion and porosity
	3	15.17	16.33	297.9 ± 9.6/320.7	Severe porosity
	4	15.17	15.27	297.9 ± 9.6/299.8	Severe slag inclusion and porosity
	5	15.17	15.32	297.9 ± 9.6/286.4	Severe slag inclusion and porosity
Modified product (New spacer frame/old clamp)	6	15.17	16.01	297.9 ± 9.6/300.76	When the old clamp breaks first, there are no obvious defects on the cross-section of the spacer frame.
Modified product (New spacer frame/new clamp)	7	15.17	17.12	297.9 ± 9.6/305.4	When the old clamp breaks first, there are no obvious defects on the cross-section of the spacer frame

## Data Availability

The original contributions presented in this study are included in the article. Further inquiries can be directed to the corresponding author.

## Nomenclature

UHV	Ultra High Vacuum
EFL	Effective flow length
LPDC	Low Pressure Die Casting
HPDC	High-pressure die casting
P	Centripetal force
n	The number of subwires
Icc	The short-circuit current
H	The tension of the subconductor
S	The diameter of the subconductor splitting circle
D	The diameter of the subconductor
SC	Squeeze Casting
